# Case report: severe heat stroke with multiple organ dysfunction – a novel intravascular treatment approach

**DOI:** 10.1186/cc3771

**Published:** 2005-07-20

**Authors:** Gregor Broessner, Ronny Beer, Gerhard Franz, Peter Lackner, Klaus Engelhardt, Christian Brenneis, Bettina Pfausler, Erich Schmutzhard

**Affiliations:** 1 Resident, Department of Neurology, Neurological Intensive Care Unit, Medical University, Innsbruck, Austria; 2 Assistant Professor, Department of Neurology, Neurological Intensive Care Unit, Medical University, Innsbruck, Austria; 3 Assistant Professor, Department of Neurology, Neurological Intensive Care Unit, Medical University, Innsbruck, Austria; 4 Professor and Chairman, Department of Neurology, Neurological Intensive Care Unit, Medical University, Innsbruck, Austria

## Abstract

**Introduction:**

We report the case of a patient who developed a severe post-exertional heat stroke with consecutive multiple organ dysfunction resistant to conventional antipyretic treatment, necessitating the use of a novel endovascular device to combat hyperthermia and maintain normothermia.

**Methods:**

A 38-year-old male suffering from severe heat stroke with predominant signs and symptoms of encephalopathy requiring acute admission to an intensive care unit, was admitted to a ten-bed neurological intensive care unit of a tertiary care hospital. The patient developed consecutive multiple organ dysfunction with rhabdomyolysis, and hepatic and respiratory failure. Temperature elevation was resistant to conventional treatment measures. Aggressive intensive care treatment included forced diuresis and endovascular cooling to combat hyperthermia and maintain normothermia.

**Results:**

Analyses of serum revealed elevation of proinflammatory cytokines (TNF alpha, IL-6), cytokines (IL-2R), anti-inflammatory cytokines (IL-4) and chemokines (IL-8) as well as signs of rhabdomyolysis and hepatic failure. Aggressive intensive care treatment as forced diuresis and endovascular cooling (CoolGard^® ^and CoolLine^®^) to combat hyperthermia and maintain normothermia were used successfully to treat this severe heat stroke.

**Conclusion:**

In this case of severe heat stroke, presenting with multiple organ dysfunction and elevation of cytokines and chemokines, which was resistant to conventional cooling therapies, endovascular cooling may have contributed significantly to the reduction of body temperature and, possibly, avoided a fatal result.

## Introduction

Heat stroke is a life-threatening disease characterized by hyperpyrexia (elevated core body temperature exceeding 40°C) and predominant central nervous system dysfunction resulting in delirium, convulsion or coma [1]. In many clinical and pathogenetic aspects, heat stroke resembles sepsis, requiring aggressive intensive care treatments, and there is growing evidence that endotoxemia and cytokines may be implicated in its pathogenesis [1-3]. We report a case of severe heat stroke with secondary multiple organ dysfunction being successfully treated with an intravascular cooling device.

## Case report

A 38-year-old male underwent a hiking tour on a hot, humid day in late July 2003. At the end of this exhausting trip he complained of dizziness, finally falling into an 'apathic' state. On the arrival of the emergency physician, the patient suffered from a generalized epileptic seizure. Subsequently, the comatose patient (Glasgow Coma Scale 6 (E 1, V 1, M 4)) developed respiratory insufficiency and cardiovascular failure (blood pressure 60/20 mmHg, heart rate 166/min). He was immediately intubated (using fentanyl (0.3 mg), etomidate (40 mg) and midazolam (20 mg)) and transported to our neurological intensive care unit (NICU).

On admission, the patient was deeply sedated and under analgesia, but still suffering from hypotension requiring immediate use of catecholamines (norepinephrine). The patient had normal weight (body mass index = 24) and no significant previous medical history. The initial cerebral computed tomography (CT) scan in combination with CT angiography did not reveal any pathologies and, to exclude an infectious origin for the central nervous system dysfunction, a lumbar puncture was carried out yielding normal cerebrospinal fluid. An initial extensive laboratory work up revealed impaired liver function (glutamic-oxaloacetic transaminase 312U/l (normal range: 10 to 50 U/l), glutamic-pyruvic transaminase 244 U/l (normal range: 10 to 50 U/l), gamma-glutamylcyclotransferase 94 U/l (normal range: 10 to 66 U/l) and a prothrombin time of 60% (normal range: 70 to 130%). Serum creatinine levels as well as blood urea nitrogen (BUN) were elevated (creatinine 2.6 mg/100 ml (normal range: 0.8 to 1.3 mg/100 ml) and BUN 30 mg/100 ml (normal range: 5 to 25 mg/100 ml)) indicating the beginning of renal failure. This situation was further complicated by rhabdomyolysis with elevation of myoglobin and creatine kinase (CK) (myoglobin peak level 33.124 μg/l (day 2), normal range: 0 to 116 μg/l) and CK peak level 102.4 U/l (day 4), normal range: 38 to 174 U/l.

**Figure 1:**
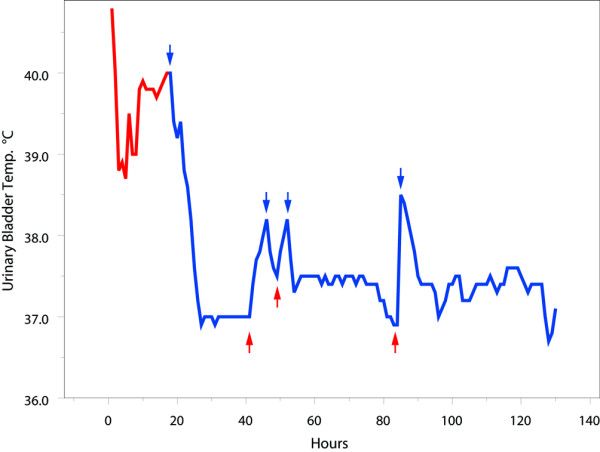
Course of core body temperature in a patient with heat stroke. The red line denotes the core body temperature while using 'conventional' temperature control methods. The blue line denotes the core body temperature while using an endovascular (CoolGard^®^) cooling treatment. Blue arrows denote the start of CoolGard^® ^treatment. Red arrows denote attempts to terminate the active cooling procedure.

At the time of admission, core body temperature measured by urinary bladder probe (Foley catheter; Kendall Curity, Mansfield, MA, USA), was 40.8°C. During the first 20 h of treatment, conventional temperature control methods including high-dose non-steroidal anti-inflammatory drugs (NSAIDs) (acetylsalicylic acid 1000 mg and paracetamol 2000 mg) and opioids (pethidine 100 mg), as well as external cooling devices such as cooling blankets (Blanketrol II^®^, Cincinnati Sub-Zero, Cincinnati, OH, USA) and Bair Hugger^® ^(Arizant Healthcare Inc, Eden Prairie, MN, USA), which were applied for 8 h, did not lead to any significant decrease in core body temperature (Figure 1). Because of subsequent deterioration of the patient's condition and insufficient conventional temperature control, an aggressive treatment approach with a novel intravascular cooling system (CoolGard 3000^® ^and Cool Line™, Alsius, Irvine, CA, USA) was begun. The heat-exchange catheter (Cool Line™) was placed into the left superior vena cava and cooled saline was infused through a closed loop system into two heat-exchange balloons located near the distal end of the catheter. The temperature of the saline solution was adjusted automatically by the CoolGard 3000^®^, which is an external temperature control unit, according to feedback to the external pump/refrigerant device from a microthermister attached to a Foley bladder catheter. Target temperature was set at 37°C for the first 25 h of intravascular treatment and subsequently at 37.5°C. Target temperature was reached within 7 h at a maximum cooling rate of 0.6°C/h and 'cooling' was prolonged at this level.

Multiple organ dysfunction and secondary rhabdomyolysis led to increased levels of myoglobin and CK (myoglobin peak level 33.124 μg/l (day 2), CK peak level 102.4 U/l (day 4)). To prevent imminent renal failure, forced diuresis was initiated and continued for 40 h using high-dose furosemide and fluids, resulting in an urinary excretion rate of more than 400 ml/h, leading to a fluid turnover of up to 24,000 ml/24 h. With this aggressive measure, we suceeded in avoiding the use of extracorporal hemofiltration and the renal parameters returned to normal values within 3 days.

Core body temperature was maintained at about 37°C and subsequently maintained at 37.5°C (± 0.2°C) with the use of the intravascular catheter over the next 5 days (Figure 1). Several attempts to stop the active cooling within this period (Figure 1) led to an immediate steep increase of core body temperature, which forced us to prolong this very efficacious endovascular treatment. Finally, after 111.5 h, CoolGard^® ^treatment was stopped, since most of the laboratory parameters had stabilized; the patient did not suffer from hyperthermia thereafter.

To confirm the diagnosis of severe heat stroke and to measure the systemic inflammatory response [2], we analyzed levels of plasma cytokines and serum chemokines 60 h after admission. The results are shown in Table 1: soluble interleukin (IL) receptor (sIL-2R) 1500 pg/ml, IL-4 3 pg/ml, IL-6 204 pg/ml, IL-8 40 pg/ml and tumor necrosis factor alpha (TNF alpha) 38 pg/ml (IL-4, IL-6, IL-8, TNF alpha analyzed by immunoenzymometric assay, Biosource, Nivelles, Belgium; IL-2R analyzed by immunoenzymometric assay, Immunotec, Marseille, France). On days 5 and 7 after admission, the values of IL-6 had decreased to 96 pg/ml (day 5) and 34 pg/ml (day 7), respectively.

Initially diagnosed aspiration pneumonia as well as sinusitis maxillaris diagnosed on the initial cerebral CT scan, were treated with tazobactam/piperacilline and clindamycin. On day 3, somatosensoric potentials did not show any pathologic results. The patient was extubated on day 8 and transferred to a regular neurological ward on day 12 with neither signs of any focal neurological nor overt cognitive deficits. At the time of discharge from the NICU, laboratory parameters had returned to normal values.

## Results and discussion

Immediate cooling and support of organ-system function are the two major therapeutic objectives in patients with heat stroke [1,3,4]. Using conventional temperature control measures such as NSAIDs or external cooling devices (cooling blankets and Bair Hugger^®^), even applied for several hours, was ineffective in combating hyperthermia in this case. So far, only one case has been reported in which a heat exchange balloon was inserted in the femoral vein [5] leading to reduction of core body temperature to 37-39°C. We succeeded in maintaining the preset normothermia (37 to 37.5°C) for more than 5 days, thus both preventing neurological sequelae and rescuing failing organ functions, in a patient with an expected mortality rate of up to 50% [1,6]. For active cooling, we used the Cool Line™ catheter placed into the upper vena cava in combination with CoolGard 3000^®^. Studies could show that this system is an efficacious tool for combating hyperthermia in patients with severe primary intracranial diseases [7-9] but has not been validated so far as a therapeutic tool in heat stroke.

The laboratory work up of chemokines (IL-8), proinflammatory cytokines (TNF alpha, IL-6), cytokines (IL-2R) and anti-inflammatory cytokines (IL-4) revealed an elevation of all parameters, which is of particular interest as it has been postulated that these cytokines and chemokines may play an important role in the pathogenesis of heat stroke [2]. In particular, the excessive elevation of IL-6 and IL-2R found in our patient is remarkable as these two markers may predict disease severity [1,2]. Considering these facts, the favorable neurologic outcome of our patient after having suffered from this 'sepsis-like syndrome' including multiple organ dysfunction, may be an indicator that intravascular cooling and maintenance of normothermia influences the inflammatory response and may lead to improved outcome in patients with heat stroke.

## Conclusion

Heat stroke is a life-threatening disease requiring immediate admission to an ICU. The progression to multiple organ dysfunction can be fatal as many organ systems may be affected. The primary therapeutic goal must be to lower the core body temperature, which may be impossible with conventional measures. In our patient, intravascular treatment was efficacious and feasible. Prospective and controlled studies comparing the efficacy of various cooling techniques in NICU patients have proven the feasibility and efficacy of this endovascular cooling device (CoolGard 3000^® ^and Cool Line™), thus it should be considered as a possible alternative to conventional treatment in heat stroke patients. In our patient, maintenance of normothermia (37 to 37.5°C) led to a favorable outcome with no neurologic impairment after the 'sepsis-like' heat stroke. Thus, further randomized and controlled studies are warranted to evaluate intravascular cooling as a possible tool in combating severe heat stroke.

## Key messages

• 		Heat stroke is a life-threatening disease requiring immediate admission to an intensive care unit.

• 		Lowering the core body temperature must be the primary goal but conventional temperature control measures, as in our case, might be insufficient in decreasing core body temperature.

• 		Intravascular cooling was efficacious and feasible in maintaining "normothermia" (37°C – 37.5°C) in our patient, leading to a favorable outcome.

• 		Intravascular cooling could be considered as a possible alternative to conventional treatment in heat stroke patients.

## Abbreviations

BUN = blood urea nitrogen; CK = creatine kinase; IL = interleukin; NICU = neurological intensive care unit; NSAIDs = non-steroidal anti-inflammatory drugs; R = receptor; TNF = tumor necrosis factor.

## Competing interests

The authors declare that they have no competing interests.

## Authors' contributions

GB and ES coordinated the data analysis and drafted the manuscript. RB, GF and BP participated in analysis of clinical data. KE and CB participated in analysis of CoolGard 3000^® ^data. PL helped to draft the manuscript. All authors read and approved the final manuscript.

## Figures and Tables

**Table 1:** Course of TNF alpha and IL levels in a patient with heat stroke

Parameters	Normal range, pg/ml	Measured values on day 3, pg/ml	Measured values on day 5, pg/ml	Measured values on day 7, pg/ml
sIL-2R	0–4.8	1500		
IL-4	NA	3		
IL-6	0–3	204	96	34
IL-8	NA	40		
TNF alpha	0–20	38		

IL, interleukin; NA, not available; TNF, tumor necrosis factor.
